# Laparoscopic Management of a Giant Echinococcal Liver Hydatid Cyst in a Technically Challenging Site With Simultaneous Ureteric Stone Removal: A Case Report

**DOI:** 10.7759/cureus.92217

**Published:** 2025-09-13

**Authors:** Qahtan A Al Dulaimi, Nour K Sabaneh, Somar Ajeka

**Affiliations:** 1 Department of General Surgery, Saqr Hospital, Emirates Health Services (EHS), Ras Al Khaimah, ARE; 2 Department of General Surgery, Ras Al Khaimah Medical and Health Sciences University, Ras Al Khaimah, ARE

**Keywords:** case report, giant hydatid liver cyst, laparoscopy technical challenges, liver hydatid cyst, multidisciplinary approach, patient-centered care

## Abstract

A 44-year-old woman presented with a two-day history of sudden, severe right upper quadrant abdominal pain. Her medical history included hypothyroidism managed with thyroxine. Eight months prior, she had undergone imaging for bilateral flank pain, which revealed a left ureteric stone with hydronephrosis (managed conservatively) and a large right hepatic lobe cystic lesion. Serologic testing later confirmed echinococcal antibodies, and the hepatic cyst was initially managed non-operatively as the patient refused surgery. The patient also reported contact with dogs, sheep, and cats in Syria one year before presentation. Repeat imaging revealed a thick-walled hepatic cyst involving segment VIII of the right lobe and segment IV of the left lobe (measuring 9x10.6x8.5 cm), along with a left distal ureteric stone.

A combined surgical approach was planned. First, the urology team performed ureteroscopic extraction of the stone with stent placement. This was followed by laparoscopic deroofing of the hepatic hydatid cyst. Intraoperatively, the cyst was aspirated and irrigated with 20% hypertonic saline, and then de-roofed, and the germinal layer was removed, followed by a second hypertonic saline irrigation. The cavity was covered with an omental flap to reduce the risk of bile leakage and bleeding. Histopathology confirmed an echinococcal cyst. Postoperatively, the patient received albendazole and recovered uneventfully. She was discharged on postoperative day four with instructions to complete two additional albendazole courses. The location of the hydatid cyst posed a significant technical challenge, as it was situated at the hepatic dome with associated liver enlargement, leaving minimal to no space between the liver and diaphragm for safe manipulation. This case highlights the importance of surgical expertise in managing hepatic hydatid disease, particularly in low-prevalence regions where specialists and resident surgeons may have limited exposure to such cases. In addition, we encourage the use of a multidisciplinary approach in surgical planning, particularly for patients with multiple surgical pathologies, for better patient-centered outcomes.

## Introduction

Hydatid cyst disease is a parasitic infection caused by the tapeworm *Echinococcus granulosus*. The larval form of this parasite can develop in various organs, most commonly the liver (70%) and lungs (20%), forming cystic fluid-filled structures known as hydatid cysts. Hydatid disease of the liver is endemic in the Middle East [1], with a higher incidence in rural and developing countries (low socioeconomic and cultural levels). The cysts are usually asymptomatic unless they grow large enough to affect nearby structures or rupture, which can result in severe complications such as intrabiliary rupture, intraperitoneal rupture, jaundice, liver abscess, or even anaphylaxis [2]. For the diagnosis of liver hydatid cysts, the IgG-ELISA (enzyme-linked immunosorbent assay) is considered the gold standard serological test. However, seronegativity may still take place during both the early and late stages of the disease [3,4]. Moreover, imaging plays an important role in diagnosis, with ultrasonography as the first-line diagnostic modality and abdominal computed tomography as the second-line diagnostic modality in cases where ultrasonography is not enough. Together, these tools are crucial for hydatid cyst classification and pre-surgical planning [5]. Globally, it is estimated that 2-3 million people are affected, even though the true burden is most probably higher due to under-reported epidemiological data. Documented incidence rates can reach 50 per 100,000 person/years, with prevalence as high as 10% in highly endemic areas [4]. The incidence of hydatid disease in the Middle East has been increasing. A study found a 459% rise in the incidence of human cystic echinococcosis in the United Arab Emirates (UAE) from 1990 to 2019 (incidence number = 34 vs 189 respectively), though the number of cases remains relatively low compared to other Middle Eastern countries [6].

This highlights the need for specialists and resident surgeons in low-prevalence regions, like the UAE, to be aware of the technical challenges that may arise during laparoscopic removal of giant liver hydatid cysts, especially in terms of accessibility to the cyst and avoiding spillage of cyst contents. We also aim to encourage the use of a multidisciplinary approach when faced with such cases, as it helps with less hospital exposure and improved patient-centered outcomes.

## Case presentation

A 44-year-old woman presented to our emergency department with severe, sudden right upper quadrant abdominal pain that had persisted for two days. The pain was not associated with nausea, vomiting, diarrhea, fever, pruritus, urinary symptoms, or recent travel. She reported a history of intermittent mild right upper quadrant pain over the past year, unrelated to food intake. She also mentioned previous exposure to dogs, sheep, and cats on a farm in Syria approximately one year before presentation. Additionally, she has a history of chronic constipation.

**Figure 1:**
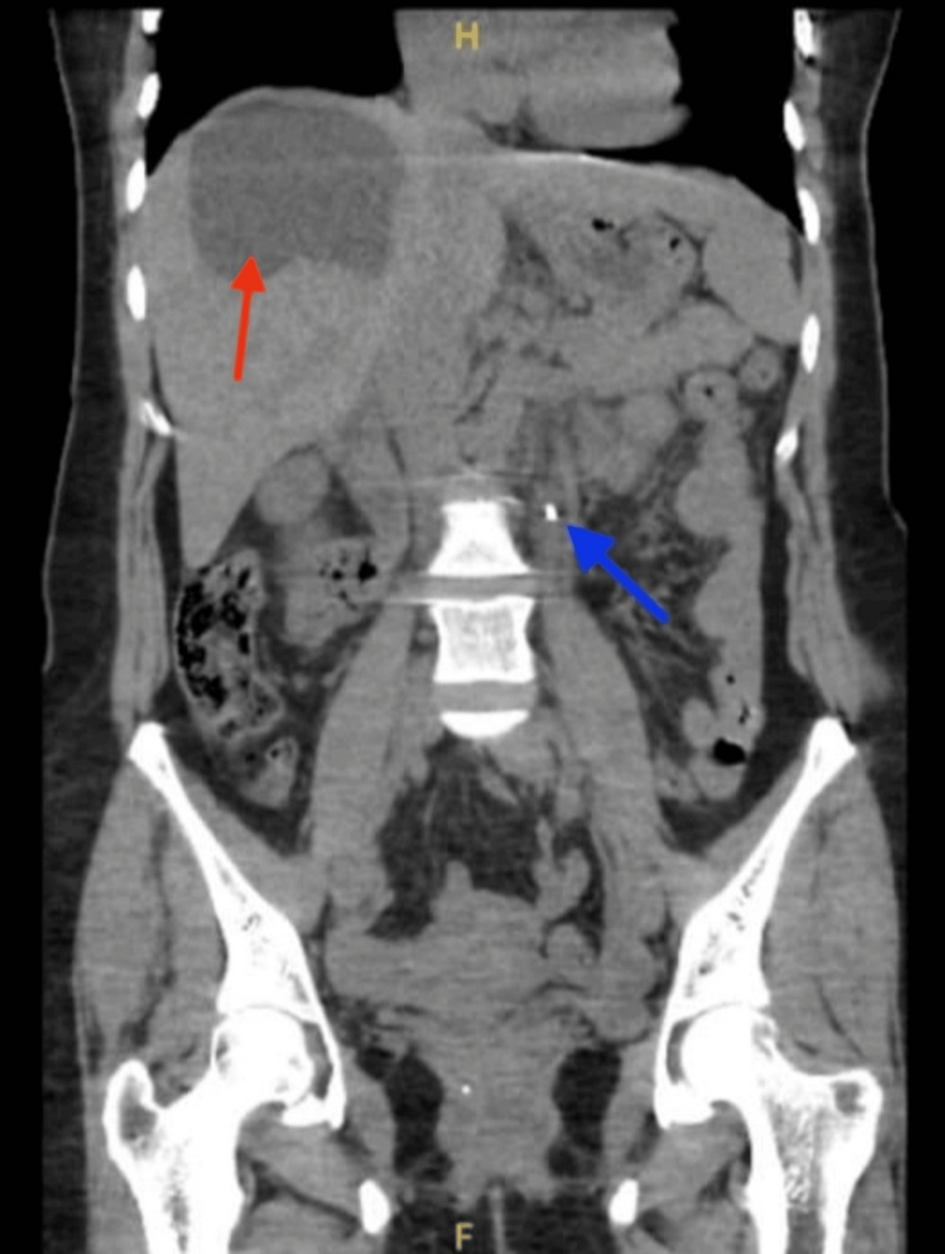
First Encounter CT-KUB (Coronal View) Showing a 5 mm Left Ureteric Stone (Blue Arrow) and Incidental Finding of a Large, Non-calcified Cystic lesion in the Right Hepatic Lobe (Red Arrow). CT-KUB: Computed Tomography of the Kidneys, Ureters, and Bladder

The patient is a known case of hypothyroidism and is maintained on daily thyroxine therapy. In addition to the current presentation, she had sought medical care eight months prior at a different hospital for bilateral flank pain of one-day duration. At that time, she also reported burning micturition and low-grade fever. Computed tomography of the kidneys, ureters, and bladder (CT-KUB) (Figure 1) revealed a 5 mm left mid-ureteric stone with mild proximal hydronephrosis. An incidental finding was a large, non-calcified cystic lesion in the right hepatic lobe with a thick wall but no daughter cells (Class IV).

The ureteric stone was managed with medical expulsion therapy. She was referred to general surgery for evaluation of the hepatic cyst, and serologic testing revealed positive echinococcus antibodies. The patient refused surgery at that time, and the hepatic cyst was therefore managed conservatively. Other than this, her medical history was unremarkable. She denied any history of smoking, alcohol consumption, or known allergies. The patient was admitted under the general surgery team and was also referred to urology.

Upon admission, the patient's vital signs were stable. Abdominal examination revealed a soft, non-distended abdomen with mild tenderness in the right upper quadrant. No masses were palpable, and there was no guarding or rigidity. Bowel sounds were audible.

Initial laboratory investigations (Table 1) revealed an elevated red blood cell (RBC) count of 5.05 × 10⁶/mcL (normal 3.80-4.80 × 10⁶/mcL), which may indicate mild dehydration secondary to ureteric stone complications. Mild hyponatremia was noted (sodium 135 mmol/L; normal 136-145 mmol/L), along with low serum osmolality (270.2 mOsm/kg; normal 275-295 mOsm/kg), suggesting electrolyte imbalance likely related to intravenous fluids administered during emergency management. The estimated glomerular filtration rate (eGFR) was 85.3 mL/min/1.73m², consistent with mild renal impairment that may have resulted from hydronephrosis. All other laboratory parameters were within normal limits.

**Table 1:** Initial Laboratory Investigations upon Admission of the Patient. ^> ^= Increased Laboratory Values ^<^ = Decreased Laboratory Values

Lab Test	Result	Reference Range
WBC	7.16 x10^3^ / mcL	4.00 - 10.00 x10^3^ / mcL
Hgb	13.80 gm/dL	12 - 15 gm/dL
Hct	42.20%	36 - 46 %
Platelet	326.00 x10^3 ^/mcL	150 - 450 x10^3 ^/mcL
Red Blood Cell (RBC) ^>^	5.05 × 10⁶/mcL	3.80 - 4.80 × 10⁶/mcL
Neutrophils	49.80%	40 - 80 %
Lymphocytes	36.30%	20 - 40 %
C-Reactive Protein (CRP)	3.1 mg/L	0.00 - 3.00 mg/L
Creatinine	74.0 umol/L	49 - 90 umol/L
Uric Acid	300 umol/L	155 - 357 umol/L
Urea Level	4.97 mmol/L	2.50 - 6.40 mmol/L
eGFR ^<^	85.3 mL/min/1.73m^2^	> 90 mL/min/1.73m^2^
Albumin	39.4 g/L	34-50 g/L
AST	20 U/L	5-34 U/L
ALT	25 U/L	4-40 U/L
Sodium (Na^+^) ^<^	135 mmol/L	136-145 mmol/L
Potassium	4.29 mmol/L	3.50-5.10 mmol/L
Osmolality ^<^	270.2 mOsm/kg	275 - 295 mOsm/kg

**Figure 2:**
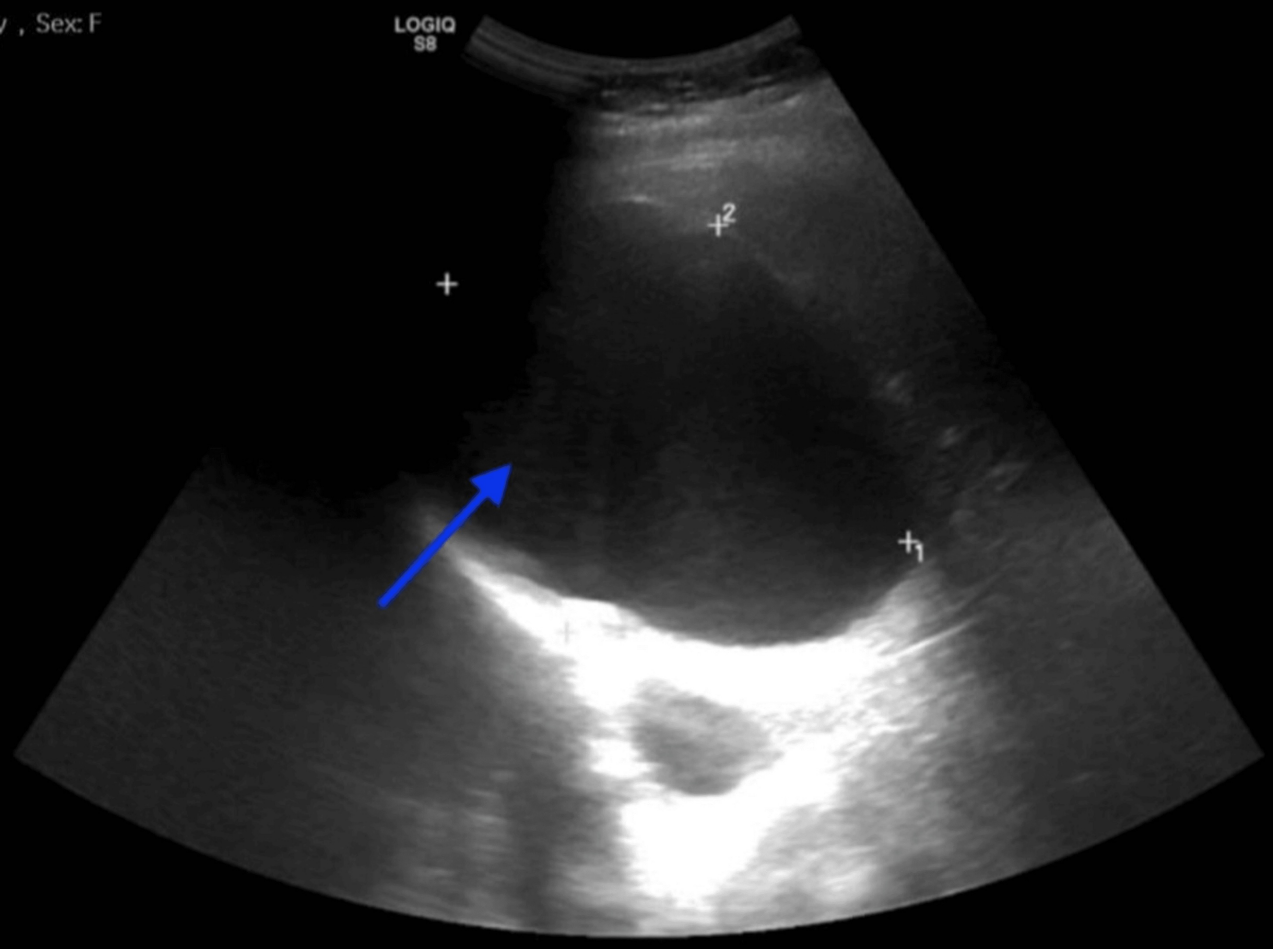
Abdominal Ultrasound Showing Hepatomegaly of the Right lobe of the Liver Especially Segment VIII with a Sizable Simple Cystic Lesion Measuring 9.7×7.9×7.3 cm (Blue Arrow).

An abdominal ultrasound (Figure 2) performed at admission revealed hepatomegaly involving the right lobe of the liver, particularly segment VIII, with a sizable simple cystic lesion measuring 9.7×7.9×7.3 cm. Mild left renal hydronephrosis was also noted.

**Figure 3:**
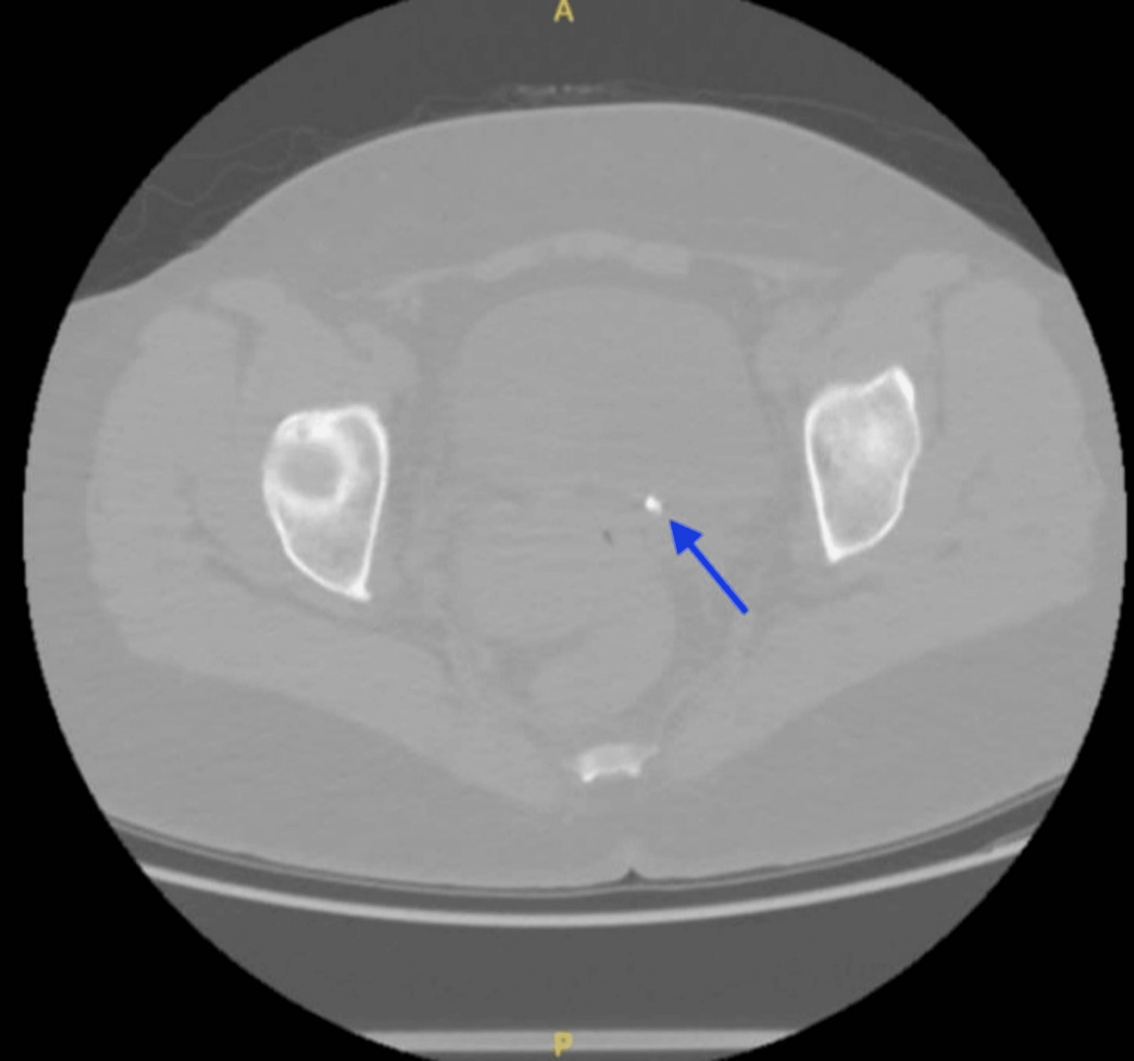
Second Encounter CT-KUB (Axial View) Showing a 6mm left Vesicoureteral Junction Stone (Blue Arrow). CT-KUB: Computed Tomography of the Kidneys, Ureters, and Bladder

As a follow-up to the CT-KUB done eight months earlier, a repeat CT-KUB was performed (Figure 3). It showed the same left ureteric stone, now at the vesicoureteral junction and measuring 6 mm, with persistent proximal left hydronephrosis above the stone. The large hepatic cystic lesion appeared to have remained largely unchanged.

**Figure 4:**
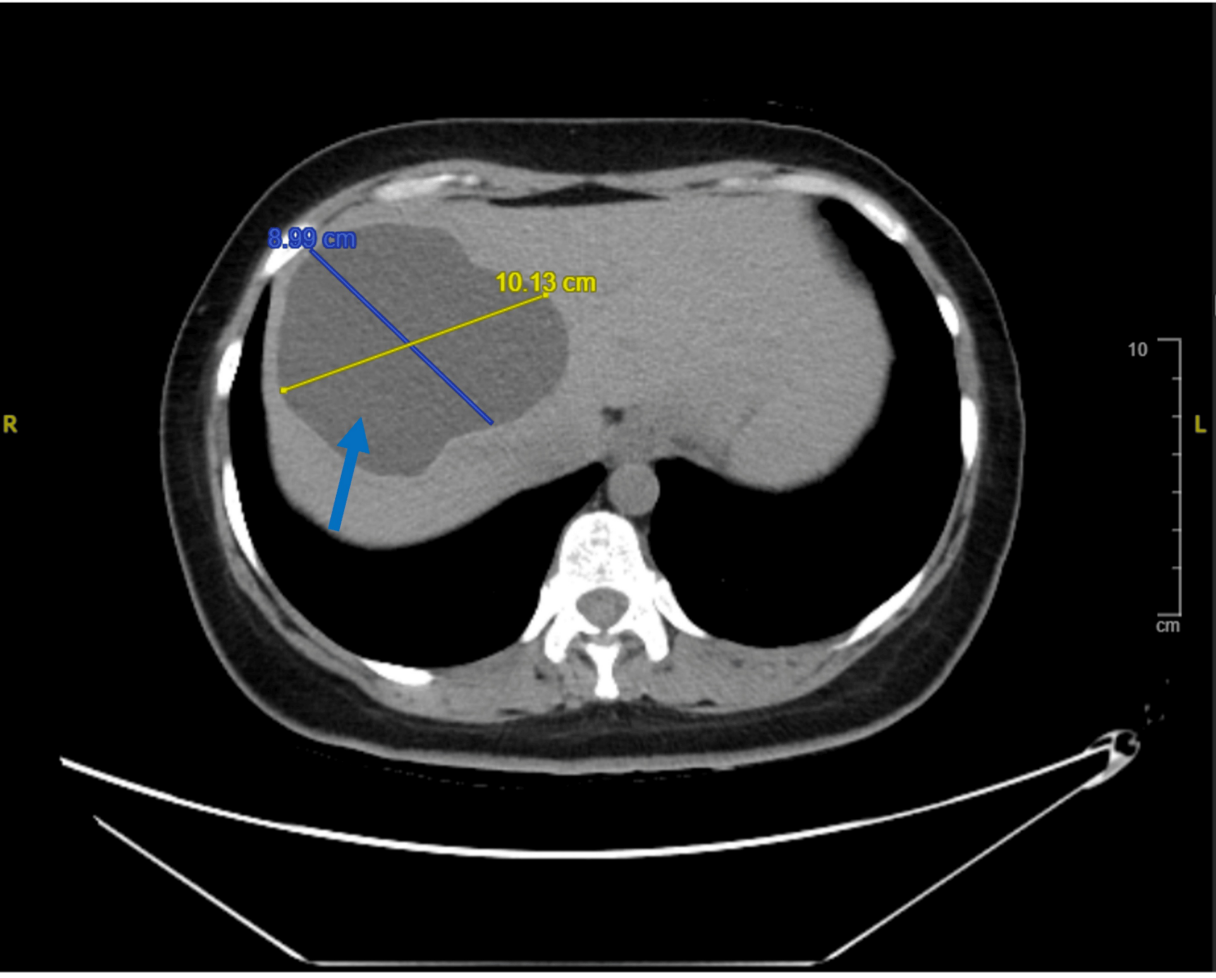
Axial View of a Non-enhanced Abdominal Computed Tomography Scan. Axial view of a non-enhanced abdominal computed tomography scan showing hepatomegaly with a large right hepatic lobe thick-walled fluid-filled hypodense cystic lesion (blue arrow).

**Figure 5:**
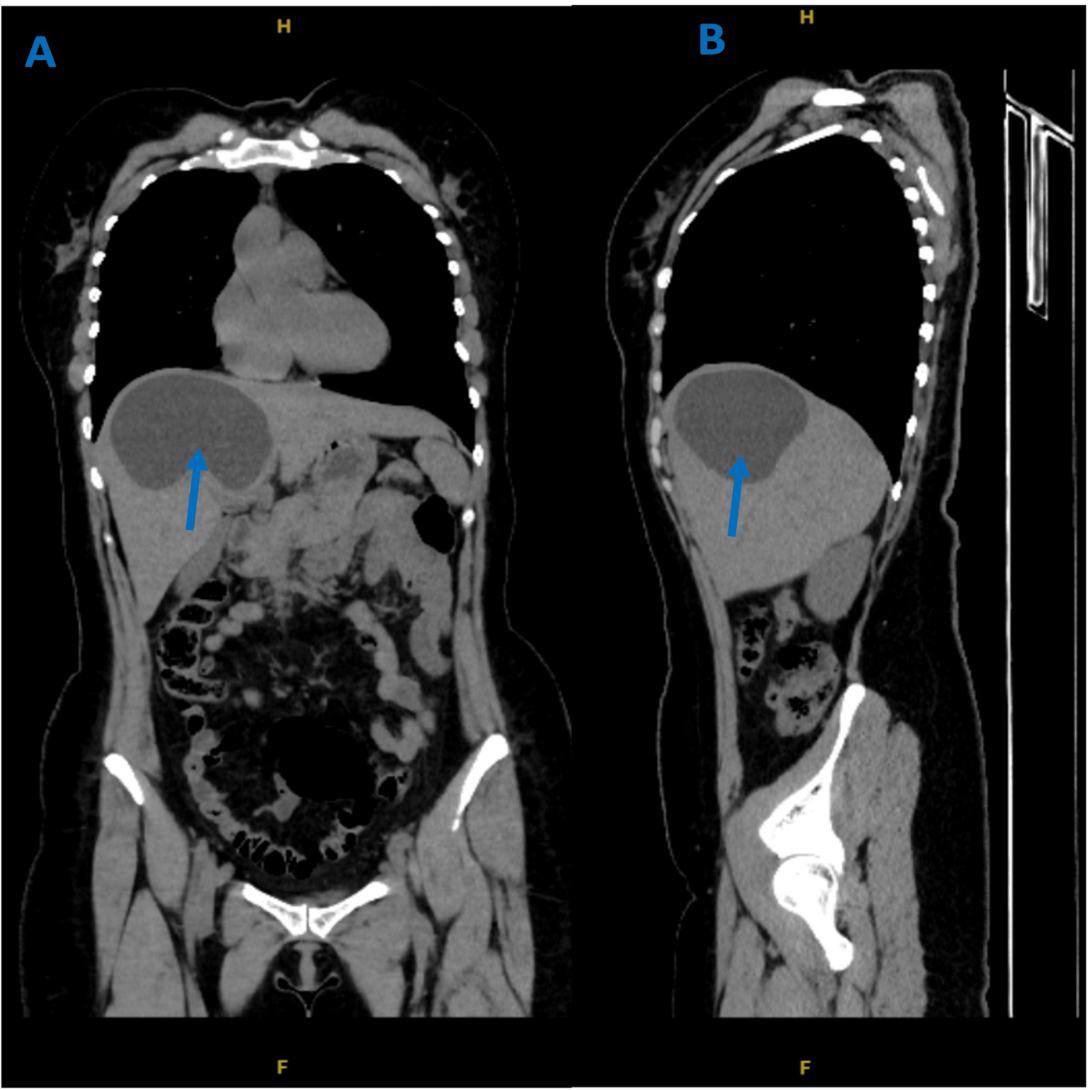
(A) Coronal View of Non-Enhanced Abdominal CT scan and (B) Sagittal View of Non-Enhanced Abdominal CT scan Both scans show hepatomegaly with right hepatic lobe segment VIII and adjoining left hepatic lobe segment IV, a large thick-walled fluid-filled hypodense cystic lesion (blue arrows). CT: Computed Tomography.

**Figure 6:**
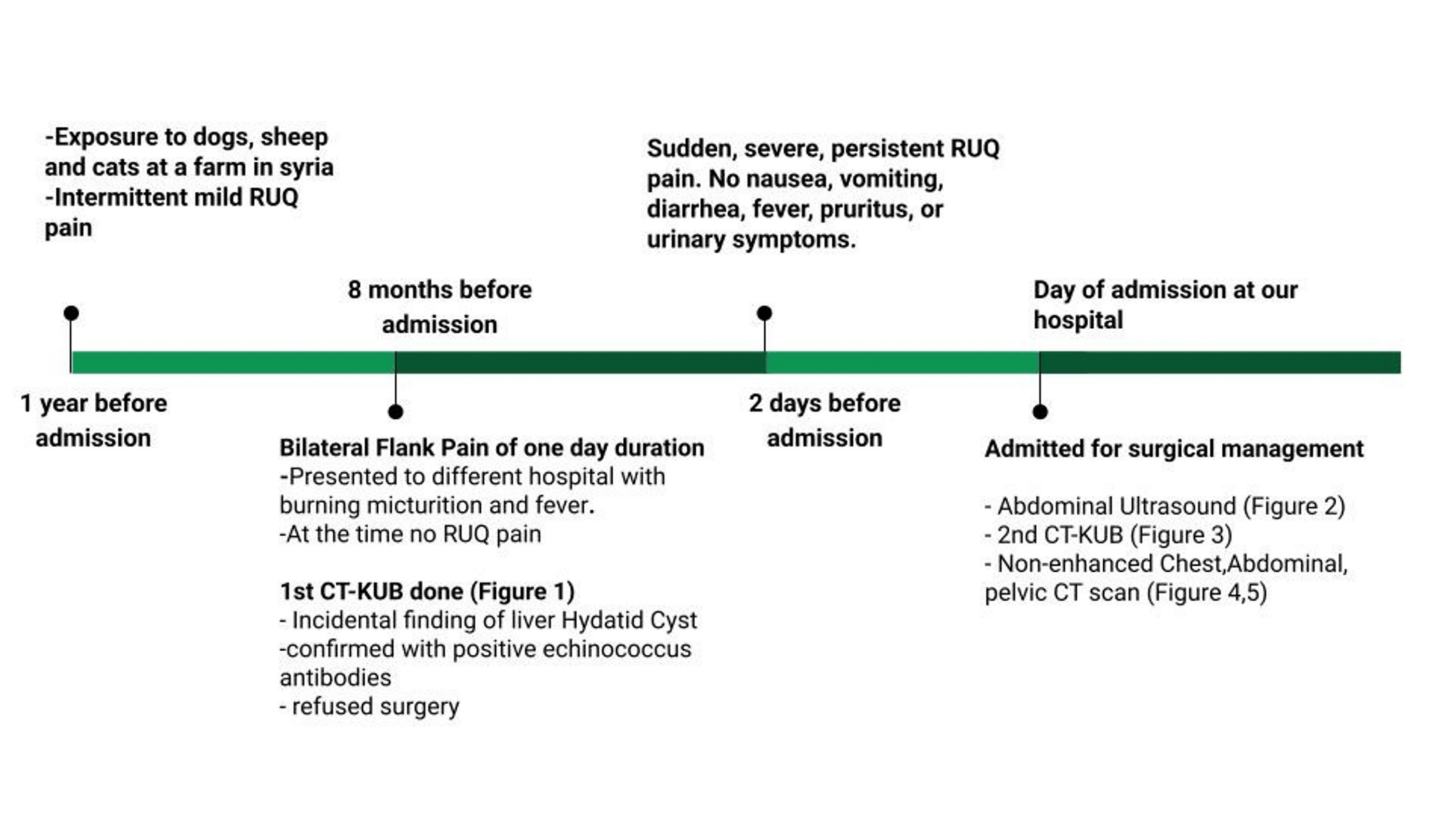
A Summary of the Chronological Progression of Symptoms and Investigations. RUQ: Right Upper Quadrant; CT-KUB: Computed Tomography of Kidneys, Ureters and Bladder; CT: Computed Tomography

A non-enhanced CT of the chest, abdomen, and pelvis revealed hepatomegaly and a large, thick-walled fluid fluid-filled hypodense cystic lesion involving segment VIII of the right hepatic lobe and the adjoining segment IV of the left lobe. The lesion measured 9x10.6x8.5 cm, with an estimated volume of 421 mL (at maximum dimension), and was closely related to the right and middle hepatic veins. Additionally, a smaller similar cyst was seen just caudal to the larger hepatic cyst (Figures 4, 5). A left distal ureteric stone with associated mild to moderate proximal hydronephrosis was also noted. A summary of the chronological progression of symptoms and investigations is provided in Figure 6.

The patient was scheduled for a single-session operation, involving two procedures. The first procedure was the removal of the ureteric stone via ureteroscopy, performed by the urology team. The second procedure was laparoscopic cyst deroofing of the liver hydatid cyst, performed by the general surgery team.

Under general anesthesia, the urology team performed rigid left ureteroscopy with basket extraction of the stone, followed by insertion of a Double J stent and a Foley catheter. Upon completion, the general surgery team proceeded with the second procedure.

**Figure 7:**
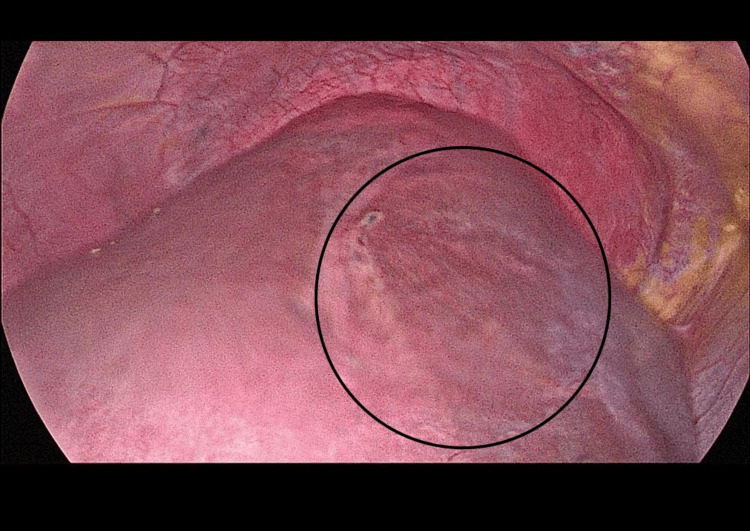
Intraoperative Finding of the Liver Hydatid Cyst (Circled).

**Figure 8:**
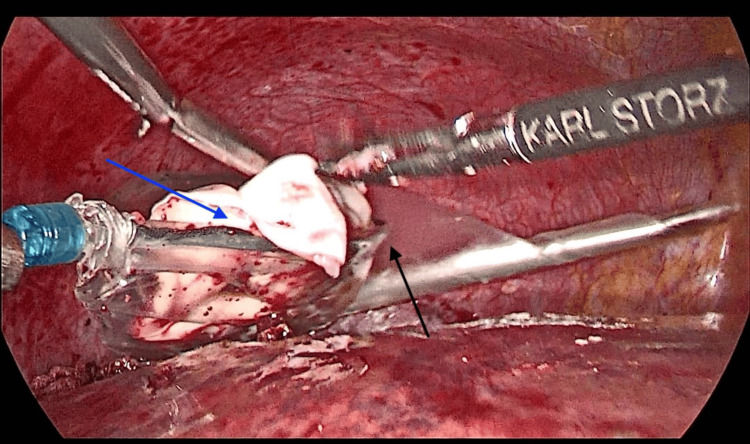
Intraoperative Cyst Deroofing. An EndoBag retriever bag (black arrow) was used to completely extract the deroofed hydatid cyst (blue arrow).

**Figure 9:**
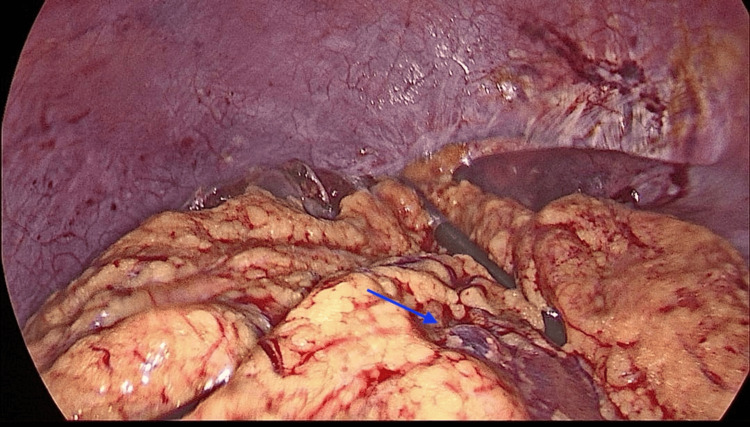
Omentoplasty. After removal of the cyst, an adjoining omental flap was used to cover the cystic cavity (blue arrow).

Intraoperatively, a clear bulge was observed in segment VIII of the liver, located at the dome on the superior surface of the liver, under the vault of the diaphragmatic surface (Figure 7). Approximately 300 mL of clear fluid was aspirated from the cyst using needle aspiration, resulting in the collapse of the bulging cyst. The cyst cavity was then injected with 300 mL of 20% hypertonic saline and left in place for 15 minutes before being re-aspirated. Deroofing of the cyst was done, and a large white layer (Germinal Layer) was removed (Figure 8). The cavity was re-irrigated with 20% hypertonic saline. An omental flap was then placed over the cyst cavity and secured to the pericystic wall with sutures, without tension (Figure 9). Two drains were placed, one adjacent to the cyst cavity and the other beneath the liver. The operation was uneventful, with no intraoperative or early postoperative complications. The germinal layer and a sample from the aspirated fluid were sent for histopathological testing, which later confirmed the cyst is of echinococcal origin.

Postoperatively, the patient was started on anti-helminthic medication albendazole at a dose of 10 mg/Kg, administered in divided doses. She complained of right-sided abdominal pain on breathing, for which incentive spirometry and analgesia were given. The sub-hepatic surgical drain drained serous fluid, while the pelvic surgical drain showed minimal fluid output. The pelvic surgical drain was subsequently removed, and the sub-hepatic surgical drain was kept in place until discharge. The Foley catheter was also removed without complications. The patient was discharged four days after the operation, with no further complaints or complications. At discharge, she was advised to continue albendazole therapy for two four-week courses, with a two-week gap between each course. At one-year follow-up, the patient reported no complaints or complications. 

## Discussion

Hydatid disease is a parasitic infection caused by Echinococcus granulosus, commonly seen in the Middle East, particularly in rural and developing regions. While the overall prevalence remains relatively low in the United Arab Emirates (UAE) compared to other Middle Eastern countries, the nation’s diverse population and exposure patterns have contributed to a noticeable increase in incidence [6]. However, due to limited clinical exposure to such cases, there are diagnostic and surgical challenges.

The liver is the most frequently affected organ, especially the right lobe, due to its rich vascular supply [5]. Most patients remain asymptomatic, and the disease is often diagnosed incidentally. However, a high index of suspicion should be maintained in individuals with a history of exposure in endemic regions, such as farm settings with dogs and sheep [7]. Human transmission occurs through the ingestion of parasite eggs, which can be acquired via direct contact with infected dogs (e.g., kissing or being licked) as their fur can be contaminated or by consuming contaminated food, water, or soil [8].

In our case, the patient’s liver hydatid cyst was discovered incidentally during evaluation for bilateral flank pain. She had a significant exposure history during her stay on a farm in Syria, where she had close contact with dogs and sheep and their surroundings, which raised clinical suspicion. The patient later presented with sudden, severe right upper quadrant pain, suggestive of an impending complication. At this point, timely diagnosis and surgical intervention are crucial, as complications such as cyst rupture can result in life-threatening complications like pleural contamination by cysto-diaphragmatic fistula due to diaphragmatic proximity, thrombosis of the right and middle hepatic veins, anaphylactic shock, or diffuse peritonitis [9].

Given the risk of rupture, the cyst’s size and its location in segments VIII and IV (close to the hepatic dome and adjacent to the right and middle hepatic veins), surgical intervention was indicated. Despite the technical difficulty, a laparoscopic approach was chosen with 30 30-degree angle, based on growing evidence supporting its safety and efficacy when performed by experienced surgeons with careful planning [10].

The cyst’s location in hepatic segment VIII, with extension into segment IV, posed several intraoperative challenges. Its adherence to the diaphragm limited working space, especially when using straight laparoscopic instruments, and increased the risk of pleural injury. More critically, the cyst was very close to the right and middle hepatic veins, which made dissection in this area potentially life-threatening. Any vascular injury could lead to significant bleeding and hemodynamic instability [11].

Several intraoperative strategies were applied to minimize spillage and complications. First, the cyst was aspirated to decompress its contents, followed by injecting 20% hypertonic saline for 15 minutes to inactivate the scolices, then aspirated again. The cyst was carefully de-roofed, and the germinal membrane was then precisely removed and collected using a retriever bag, avoiding contamination. The cavity was then re-treated with the 20% hypertonic saline to ensure full scolicidal effect. The World Health Organization Informal Working Group on Echinococcus (WHO-IWGE) recommends the use of 20% hypertonic saline for its scolicidal effect in both open and laparoscopic surgeries for hydatid cyst removal. This concentration works by creating an osmotic gradient, which dehydrates and destroys the protoscolices without causing excessive tissue toxicity. In contrast, for minimally invasive procedures like PAIR, 20% hypertonic saline and 95% ethyl alcohol can be used as recommended [12]. 

To manage the residual cavity, an omental flap with good blood supply was applied. The omentum’s absorptive, adherent, macrophage-stimulating, and angiogenic properties make it an ideal option, helping to seal the cavity and reduce the risk of postoperative complications such as biliary leakage or fistulas, surgical site infections, or deep cavity abscess formation [10,13].

Laparoscopy has favorable outcomes when compared to open surgery. Patients benefit from reduced postoperative pain, risk of wound infection, short hospital stay, and faster recovery. [10,14]. Adequate knowledge of liver anatomy, expertise, and thoughtful preoperative planning are necessary to accomplish these benefits. In low-prevalence regions like the UAE, such cases may present diagnostic and technical challenges, making multidisciplinary planning and surgical preparedness essential.

An important aspect of this case was the decision-making in managing the hepatic hydatid cyst and the ureteric stone in a single surgical session. This approach limited the need for multiple hospital admissions, longer hospital stay and anesthesia exposure. It aligns well with recommendations for multidisciplinary, efficient, cost-effective, patient-centered care in post-COVID settings. It also reduced the psychological load on the patient, going through two separate operations. This approach demonstrates the importance of coordinated communication between different departments in surgical planning, especially when dealing with sensitive and complex cases.

## Conclusions

This case highlights the challenges faced in managing hepatic hydatid disease in low-prevalence regions. The successful laparoscopic approach reflects the feasibility of minimally invasive techniques even in anatomically challenging locations, given that appropriate expertise and multidisciplinary coordination are available. A multidisciplinary surgical approach is encouraged in the presence of multiple pathologies. Single operation for two different procedures reduces the need for multiple hospital admissions, longer hospital stay, and anesthesia exposure. The strategy should focus on patient-centered outcomes.
